# Phenolic screening, antioxidant activity and diuretic effect of Moroccan *Pinus pinaster* Bark extract 

**DOI:** 10.22038/ajp.2024.25198

**Published:** 2025

**Authors:** Widad Tbatou, Abderrazak Aboulghazi, Asmae El Ghouizi, Karima El-Yagoubi, Najoua Soulo, Zineb Benziane Ouaritini, Badiaa Lyoussi

**Affiliations:** 1 *Laboratory of Natural Substances, Pharmacology, Environment, Modeling, Health, and Quality of Life * *(SNAMOPEQ), Faculty of Sciences Dhar El Mahraz, University Sidi Mohamed Ben Abdellah, Fez, 30000, Morocco*; 2 *The Higher Institute of Nursing Professions and Health Techniques (ISPITS), Fez, 30000, Morocco*

**Keywords:** Pinus pinaster, Phytochemicals, Diuretics, Antioxidants

## Abstract

**Objective:**

This study aims to evaluate the chemical composition antioxidant activity, and diuretic effects of Moroccan *Pinus pinaster* bark ethanolic extract (PPBE).

**Materials and Methods:**

The phytochemical composition of PPBE was assessed using HPLC-DAD. Total polyphenols and flavonoids were quantified using the Folin-Ciocalteu and aluminum trichloride methods, respectively, while mineral content was determined by plasma mass spectrometry. Antioxidant activity was assessed using the reducing power assay, total antioxidant capacity, and anti-DPPH free radical assay. For the diuretic effect, sixteen male Wistar rats were divided into four groups: control (distilled water, 10 ml/kg of BW), furosemide (10 mg/kg of BW), and PPBE (200 and 400 mg/kg of BW) groups. After 15 days, plasma and urine were collected for creatinine, potassium, and sodium analysis, along with urine output measurement. Statistical analysis employed one-way ANOVA followed by Tukey multiple comparison test.

**Results:**

The PPBE displayed high phenolic content and potent antioxidant properties. Besides, the PPBE phenolic screening showed nine phenolic compounds with ferulate glucoside, gallic acid, and catechin as the main compounds. The PPBE demonstrated a richness in essential minerals. Furthermore, at both doses (200 and 400 mg/kg) PPBE led to a notable elevation in urine flow, urinary sodium concentration, and creatinine clearance, without affecting plasma electrolytes. In contrast, furosemide caused a reduction in plasma potassium levels.

**Conclusion:**

PPBE could serve as a bioactive component, antioxidant, or preservative in food formulation. Moreover, it exhibits a diuretic effect without altering plasma composition.

## Introduction

Forests are considered the primary source of medicinal plants and forest products (Ashmawy et al., 2020), producing large amounts of solid waste containing valuable substances. In this regard, there is a significant deficit in converting this waste into potentially innovative products that could generate more revenue compared to conventional commercial applications, considering sustainability and positive health effects (Klimánkova, 2007). Currently, a wide range of illnesses can be linked to the intensified generation of free radicals and the resulting oxidative stress. In addition, it was revealed that oxidative stress and inflammation are contributors to kidney disease, potentially exacerbating further harm to the kidneys (Tucker et al., 2015). Consequently, there is a growing interest in discovering and promoting novel ingredients possessing antioxidant properties to avoid or prevent excessive oxidation (Willcox et al., 2004). Various compounds present in plants hold promise for use as nutraceuticals, notably phenolic substances, a diverse class of molecules exhibiting a broad spectrum of bioactive proprieties (Yesil Celiktas et al., 2010). These include antioxidant, anti-inflammatory (Hajhashemi et al., 2021), anti-atherogenic, antibacterial (Ferreira-Santos et al., 2020), neuroprotective (Mansouri et al., 2021), and diuretic properties (Santos et al., 2021).

Diuretics, whether used independently or in conjunction with other medications, play a crucial role in treating conditions such as hypertension, congestive heart failure, ascites, and pulmonary edema (Lahlou et al., 2007; Roush et al., 2014). These diuretics enhance the excretion of water and salt (sodium) through urine, leading to a reduction in blood pressure and a decrease in blood volume (Ntchapda et al., 2016; Wile, 2012). Two commonly employed diuretics, thiazides and the high-ceiling loop diuretic furosemide, have been linked to various adverse effects and cannot be administered in certain physiological cases. These include electrolyte imbalance, metabolic alterations, the onset of new diabetes, activation of the renin-angiotensin-neuroendocrine systems, and impairment of sexual function (Gupta and Neyses, 2005). The exploitation of plant by-products represents a fascinating potential for the discovery of new substances with a diuretic effect, if we consider that each of these plant by-products can contain hundreds or even thousands of secondary metabolites. The identification and purification of these molecules can bring interesting therapeutic interest to various pathologies.

In this sense, bark, or peel, is the outer layer of trees, bushes, plants, and some fruits. *Pinus pinaster* bark serves as a compelling reservoir of novel biomedical compounds and displays the highest content of bioactive compounds as compared to other commonly studied plant species, making it a promising candidate for therapeutic applications. (Mármol et al., 2019a). Until just a few years ago, it was commonly dismissed as a byproduct in the wood industry, with minimal applications such as fuel or as a substrate in gardening. Among its constituents are substances with diverse functions, including the ability to delay or prevent pathogen invasion storing reserve substances and plant hormones (Mármol et al., 2019b). 

*Pinus* is recognized as the largest genus within the Pinaceae family, encompassing over 109 distinct species recognized by Farjon (2001) (Plomion et al., 2007). The *Pinus* genus has been extensively utilized in traditional medicine due to its various pharmacological attributes, including anti-aging and anti-inflammatory benefits. Additionally, there is potential for their use in addressing conditions such as liver diseases, skin disorders, and hypertension (Xie et al., 2015). 

*Pinus pinaster (P. pinaster)*, also known as* maritime pine, *is predominantly used for nutritional and pharmaceutical applications. *P. pinaster* is found in regions such as Spain, the southern part of France, Italy, Portugal, and Morocco, with smaller populations in Malta and the northern part of Iran*. P. pinaster* sub-species differ in their cold and salt tolerance, as well as their terpenes and protein content (Rohdewald, 2002).

The bark of *P. pinaster* is a great source of phenols (Jerez et al., 2006). *P. pinaster* bark has been cited in the scientific literature for many possible applications. It has been reported that its extracts possess antioxidant, antiradical, and anti-inflammatory activities and are distributed as a nutritional supplement and an herbal-based medication, Pycnogenol® (Chupin et al., 2013).

The purpose of this study is to conduct a thorough chemical analysis of the phenolic compounds and mineral content present in extracts derived from the bark of *P. pinaster* utilizing a water-ethanol solvent. Additionally, the research aims to investigate the antioxidant potential and examine the diuretic effects of these extracts.

## Materials and Methods

### Bark origin and preparation

The *P. pinaster* bark was gathered in Sefrou (33°49’49.886’’N, 4°50’7.134’’W), Morocco. This plant was chosen for its many therapeutic properties. The bark was cleaned with water for 10 min, then washed twice with distilled water and left to air-dry in an oven at 40°C for two days (Ferreira-Santos et al., 2020). It was then crushed, reduced to powder, and stored in hermetically sealed bottles in a dry place away from humidity and light. The plant powder obtained was then used for extraction.

### Pine bark extract

To do a traditional maceration at room temperature for 72 hr under agitation for 24 hr, a total of 20 g of *P. pinaster* bark was contacted with 200 ml of ethanol/water (70-30% v/v) in 500 ml flasks. After extraction, the extract was vacuum-filtered using Whatman paper using a Buchner funnel. The filtrate was rotary-evaporated at 40°C under vacuum to remove ethanol. The filtrate was gathered and dried (Royer et al., 2013). The powder obtained was *P. pinaster* bark ethanolic crude extract (PPBE).

### Animals

Sixteen male Wistar rats (172.62±14.62 g), were used to achieve this experiment. The breeding of these animals is done in the animal house of Sidi Mohamed Ben Abdallah University. Indeed, they were housed in cages where food and tap water were freely available, under ambient temperature conditions (25°C), with a natural cycle of 12 hr of light followed by 12 hr of darkness. The research was conducted by ethical guidelines for the use and care of laboratory animals, and approval from the Ethical Committee, Faculty of Sciences, Fez, Morocco, was obtained (USMBA-SNAMOPEQ 2017-03). All efforts were made to minimize animal suffering and the number of animals used.

### Experimental design

The diuretic activity was evaluated following the protocol described by El Ghouizi et al. (El Ghouizi et al., 2020). Before beginning the experiment, each rat was put into a metabolic cage for 48 hr. Four groups (n=4) were employed and subjected to a 12-hour food deprivation period before testing, with access to water. 

The control group (Group 1) received 10 ml/kg body weight of distilled water orally via gavage. Group 2 was administered with 10 mg/kg body weight of furosemide orally via gavage. Group 3 received 200 mg/kg body weight of PPBE, while Group 4 received a higher dose of 400 mg/kg body weight of PPBE. 

The furosemide, a widely recognized loop diuretic, was employed as a standard reference to evaluate and compare the diuretic effects of the PPBE extract, and the extract doses were chosen based on the study conducted by Hajhashemi et al. (2021). On treatment days 0 and 15, urinary samples were collected using graduated cylinders to quantify water excretion. Subsequently, the samples underwent filtration, centrifugation, and preservation for the analysis of sodium, potassium, and creatinine levels. 

The animals were anesthetized using diethyl ether, and blood samples were collected from the retro-orbital plexus using heparinized capillary tubes at the end of the treatment (El Ghouizi et al., 2020). After that, the plasma was separated by centrifugation at 10,000 × g for 10 min, and it was kept at -20°C so that creatinine, sodium, and potassium could be measured (Bakour et al., 2017). On day 15, creatinine clearance was also computed using the following formula:

$\mathrm{Creatinine clearance}(\mathrm{ml}/\min)=\left(\frac{\mathrm{Ucrea}}{\mathrm{Pcrea}}\right)\mathrm{*V}$

Ucrea: Urinary creatinine concentration

Pcrea: Plasma creatinine concentration

V: Urinary volume (ml/min)

Using the following formula, the osmolar clearance (Cosm) was also determined from urine volume (V), 

plasma osmolarity (Posm), and urinary osmolarity (Uosm): Cosm = (V × Uosm) × 1/Posm. 

Next, the below equation was used to calculate the free water clearance (C_H2O_): 

C_H2O_ = V - Cosm. 

Free water reabsorption (T_CH2O_) was assessed using the following formula:

 T_CH2O _= −(C_H2O_). 

### High-Performance Liquid Chromatography with Diode-Array Detection Analysis

The analysis of phenolic compounds in PPBE was performed by a SHIMADZU PROMINANCE HPLC equipped with a degasser, a DAD detector, a quaternary pump type LC A20, and a thermostatically controlled automatic injector. An Agilent Zorbax C18 column (4.6 mm x 150 mm, 5 µm 100A) was used for the HPLC separation of polyphenols. The column temperature was 30°C, the injection volume was 10 µl, and the flow rate of the mobile phase was 0.25 ml/min using a ternary gradient of acetonitrile, methanol, and water acidifier. To find the response factor, solutions of gallic acid and Tyrosol standards were administered under identical circumstances (Vivar-Quintana et al., 2018).

### Quantification of total phenolic contents (TPC)

With certain modifications, the Folin-Ciocalteu reagent was used to calculate the total phenolic content (TPC) of PPBE. A volume of 500 µl of 10-fold diluted Folin-Ciocalteu reagent was mixed with 100 µl of the extract (Singleton and Rossi, 1965). Next, 400 µl of 7.5% sodium carbonate Na_2_CO_3 _was added to promote an alkaline medium for the redox reaction, then incubated at room temperature in the dark for 2 hr. The blue color produced was measured at 765 nm to estimate its absorbance at various intensities. Gallic acid equivalent (mg GAE/g DM) was used to express the data in milligrams per gram of dry matter weight. 

### Total flavonoid content (TFC)

 Flavonoid levels were measured by a method using aluminum trichloride (AlCl_3_) (Deghima et al., 2020). Here, 500 µl of the extract was combined with 500 µl of 20 mg/ml aluminum chloride (AlCl_3_) solution and kept for fifteen minutes in the darkness. Under identical conditions, a standard calibration curve was also prepared with quercetin solutions at different concentrations (1 mg/ml; 0.5; 0.25; 0.125; 0.0625; 0.0312; 0.015; 0.007; 0.003; and 0.00), the absorbance was then measured at 430 nm. The total flavonoid concentration of the extract is expressed as milligrams of quercetin equivalents per gram of dry plant matter (mg QE/g DM).

### Free radical scavenging activity (using 2,2-Diphenyl-1-picrylhydrazyl Assay )

This approach relies on assessing the capacity of antioxidants to capture the DPPH free radicals. The impact of PPBE on DPPH free radicals was evaluated using the method outlined by Jiménez-Moreno et al. (Jiménez-Moreno et al., 2019). A series of dilutions of the extract with different concentrations (1mg/ml; 0.5; 0.25; 0.125; 0.0625; 0.0312; 0.015; 0.007; 0.003; and 0.00) was prepared. Then, 150 µl of each dilution was mixed with 2.85 ml of a freshly prepared methanolic solution of DPPH (0.025 g/L). In what concerns the negative control, the latter was prepared in parallel by mixing 150 µl of the solvent used with 2.85 ml of a methanolic solution of DPPH. After incubation in darkness for 30 min and at room temperature, the absorbance readings were carried out at 515 nm using a spectrophotometer. Using the following formula (1), the IC_50_ was determined based on the graph produced by the percentage of inhibition. 

IC_50_ is equal to [(A0-A1/A0) ×100] ^(1)^, where A0 represents the absorbance of a blank sample with the same solvent and DPPH solution content as the negative control, and A1 represents the sample's absorbance. The tests were conducted in triplicate, and the mean±SD of findings are shown. 

### Reducing power assay (RP)

The reducing power assay (RP) was done using the procedure outlined by El Ghouizi et al. (2021). A mixture of 200 µl of 0.2 M sodium phosphate buffer (pH 6.6) and 200 µl of 1% potassium ferricyanide was combined with 50 µl of PPBE. After 20 min of incubation at 50°C, 200 µl of 10% trichloroacetic acid, 200 µl of distilled water, and 120 µl of 0.1% ferric chloride were added to the mixture. 700 nm was used to measure the absorbance. Based on the graph of absorbance against extract concentration in the solution, the extract concentration yielding 0.5 absorbance (EC_50_) was determined. As a positive control, ascorbic acid was employed. The tests were performed three times, and the mean ± SD of findings was reported. 

### Total antioxidant capacity assay (TAC)

The ammonium molybdate colorimetric technique, as described by El Ghouizi et al. (2021), was used to calculate the total antioxidant capacity (TAC). Briefly, 1 ml of reagent solution (0.6 M sulfuric acid, 28 mM sodium phosphate, and 4 mM ammonium molybdate) was mixed with 50 μl of PPBE. The mixture was capped and allowed to sit for 90 min at 95°C in a thermal block. The reaction mixture's absorbance was measured at 700 nm in comparison to a blank. The standard calibration used was ascorbic acid, and the findings are presented as mg of ascorbic acid equivalent/g of dry plant matter (mg EAA/g DM).

### Mineral content

The mineral composition was determined using the method described by Zuluaga et al. (2014). Briefly, each sample was burned until only ash remained, and then, 5 ml of 0.1 M nitric acid was added to the ash of each sample. Dryness was achieved by heating the mixture. The solutions were increased to 25 ml with distilled water after 10 ml of the same acid was added. Mineral elements were determined using an air/acetylene flame, and the quantitative determination was carried out after calibrating the instrument using different concentrations of Ca, Na, K, Fe, P, Zn, Mn, B, Cu, Cr, Cd, and Ni dissolved in 0.1% lanthanum using inductively coupled plasma mass spectrometry (ICP-MS). The results were expressed in mg of mineral per kilogram of *P. pinaster* bark (mg/Kg). All samples were analyzed in triplicate. 

### Statistical analysis

The data is presented as mean±SEM. The statistical analysis was performed by GraphPad Prism Software 8, using a one-way analysis of variance (ANOVA) followed by *post hoc* Tukey's multiple comparison test. A p<0.05 denoted significant differences.

## Results

### HPLC-DAD analysis

**Figure 1:**
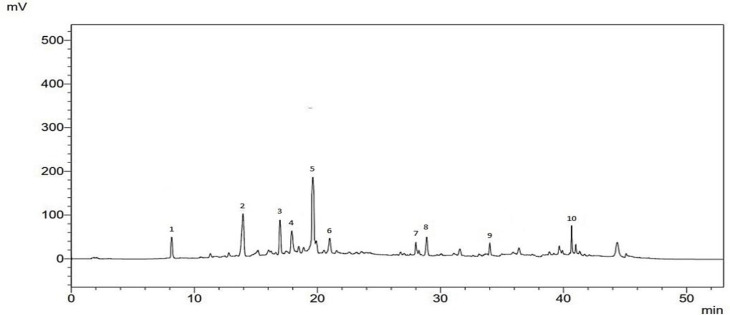
Chromatogram of ethanolic extract of P. pinaster bark (PPBE).

The determination and characterization of phenolic compounds in the PPBE were conducted using High-Performance Liquid Chromatography with Diode Array Detection (HPLC-DAD). The data is reported in Figure 1 and Table 1. According to the results, PPBE contained 9 polyphenol compounds, with the highest concentration registered for ferulate glucoside (31.07±1.45 mg/g), gallic acid (24.52±0.25 mg/g), catechin (21.02±1.38 mg/g), proanthocyanidin (14.31±0.97 mg/g), and a taxifoliol glucoside (9.1±1.27 mg/g).

**Table 1:** Phenolic compounds identification and quantification in ethanolic extract of P. pinaster bark (PPBE).

	**Phenolic compounds**	**Concentration in (mg/g)**
1	Taxofoliol glucoside	9.1±1.27
2	Gallic acid	24.52±0.25
3	Catechin	21.02±1.38
4	Epicatechin	8.9±0.56
5	Ferulate glucoside	31.07±1.45
6	Taxifoliol	5.13±1.23
7	Procyanidin B2	7.9±0.14
8	Procyanidin B3	8.33±0.22
9	Procyanidin B1	7.87±0.18
10	Proanthocyanidin	14.31±0.97

### Phenolic and flavonoid contents of PPBE

The PPBE was analyzed for total flavonoid and phenolic content through spectrophotometry. Table 2 illustrates the obtained results. The concentration of TPC in the extract was approximately 34.11±0.02 mg GAE/g DM. Additionally, the extract exhibited a TFC level of around 24.96±0.03 mg QE/g DM.

### Antioxidant activities (DPPH, RP, and TAC)

As presented in Table 2, PPBE demonstrated noteworthy antiradical DPPH activity, showing an equivalent inhibition concentration (IC_50_ value of 9.76±1.14 µg/ml) compared to the standard ascorbic acid (IC_50 _= 5.59±0.03 μg/ml). The RP assay further affirmed the potent reducing capability of PPBE, indicated by an EC_50_ of 0.037±0.004 mg/ml. The overall antioxidant capacity (TAC) was notably high, registering at 975.44±24.59 mg EAA/g DM.

### Mineral content assay

The analysis of the mineral composition of PPBE is summarized in Table 3. Notably, substantial quantities of macroelements, such as calcium (1250.42±71.30 mg/kg), magnesium (148.97±12.68 mg/kg), sodium (58,90±1.55 mg/kg), potassium (53.64±6.16 mg/kg), and phosphorus (29.73±0.38 mg/kg), were detected. The bark also exhibited appropriate levels of microelements, including iron (30.96±0.76 mg/kg), zinc (5.94±0.28 mg/kg), manganese (2.76±1.06 mg/kg), copper (0.22±0.01 mg/kg), and chromium (0.11±0.002 mg/kg). It is important to note that concentrations of toxic minerals, 

specifically, cadmium, and nickel, were determined to be below the established environmental safety limits and monthly levels of human intake.

**Table 2:** Total flavonoid content (TFC), total phenolic content (TPC), and antioxidant activity of P. pinaster bark extract (PPBE). The values are expressed in terms of means±SD of three experiments

Extract/ Standards	TPC (mg GAE/g DM)	TFC (mg QE/ g DM)	DPPH (IC_50_ µg/ ml)	FRAP (EC_50_ mg/ml)	TAC (mg EAA/g DM)
**PPBE**	34.11	24.96	9.76±1.14	0.037±0.004	975.44±24.59
**A. A**	-	-	5.59±0.03	0.031±0.07	-

*GAE: gallic acid equivalents; QE: Quercetin equivalents; DM: Dry plant matter; DPPH: 2,2-diphényl-1-picrylhydrazyle;* *IC 50: Inhibition concentration 50; FRAP: Ferric Reducing Antioxidant Power; EC*_50_ *: Effective concentration 50; TAC: Total antioxidant activity; EAA: Equivalent acid gallic; AA: Ascorbic acid*

**Table 3:** The mineral content of P. pinaster Bark expressed as mean±SD

**Mineral content**	**Concentration (mg/kg)**
	*P. pinaster* Bark
Calcium (Ca)	1250.42±71.30
Magnesium (Mg)	148.97±12.68
Sodium (Na)	58,90±1.55
Potassium (K)	53.64±6.16
Phosphorus (P)	29.73±0.38
Iron (Fe)	30.96±0.76
Zinc (Zn)	5.94±0.28
Manganese (Mn)	2.76±1.06
Copper (Cu)	0.22±0.01
Chromium (Cr)	0.11±0.002
Cadmium (Cd)	< 0.01
Nickel (Ni)	< 0.01

### Effect of daily oral administration of PPBE on urinary flow

**Figure 2:**
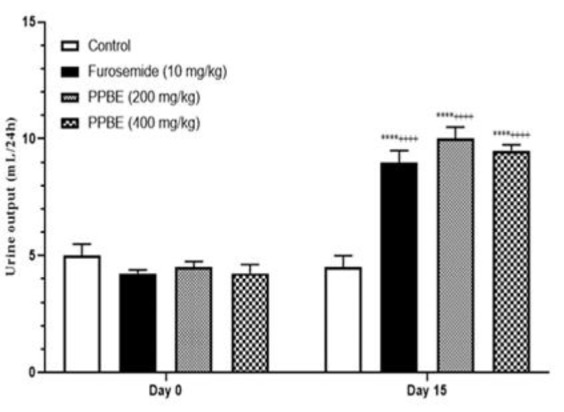
Urine output (ml/24 hr) with daily oral administration of PPBE, furosemide, and distilled water. Data are expressed as mean±SEM; PPBE D1= P. pinaster bark extract dose 1; PPBE D2= P. pinaster bark extract dose 2; *Comparison between day 0 and day 15 ****p<0.0001; +Comparison between control group and all groups in day 15++++p<0.001.# Comparison between furosemide group and PPBE ### p<0.001.

The present study evaluated urinary flow on days 0 and 15 during the treatment period. Analysis of the data presented in Figure 2 revealed that the administration of distilled water did not lead to a significant alteration in urinary flow. However, oral administration of PPBE at both doses of 200 and 400 mg/kg B.W showed a highly significant increase in urinary output as compared to the control and furosemide groups (p<0.0001). At the 200 mg/kg body weight dose, there was an elevation in urine output from 4.5±0.25 to 10±0.5 ml/24 hr. Similarly, at the higher dose of 400 mg/kg body weight, urine output increased from 4.25±0.37 to 9.5±0.25 ml/24 hr.

In comparison, the administration of furosemide also induced a substantial increase in urine output compared to the distilled water group (p<0.0001). These findings underscore the marked influence of PPBE on urinary flow, with both doses exhibiting significant effects.

### Effect of oral administration of PPBE on urinary electrolyte excretion

Table 4 outlines the effects of PPBE and furosemide on the urinary concentration of potassium and sodium. Furosemide and both PPBE doses demonstrated a significant elevation in urinary sodium concentration compared to the control group (p<0.0001), with a dose-dependent increase. At the higher dose of 400 mg/kg body weight, a substantial value of 105±0.5 mmol/l was observed, while at the lower dose of 200 mg/kg body weight, the value was 103.5±1.75 mmol/L. Furosemide exhibited an increase of 124.8±0.2 mmol/L. 

Regarding potassium, oral administration of PPBE resulted in a significant reduction in urinary potassium concentration compared to both the control and furosemide groups. As summarized in Table 4, results indicated a significant reduction in the urinary potassium elimination at both doses 200 and 400 mg /kg B.W (51±0.1 mmol/L, and 53.02±0.35 mmol/L respectively).

On the other hand, treatment with furosemide significantly increased kaliuresis to 72.1±0.1 mmol/L compared to all other groups (p<0.0001). 

**Table 4:** Urinary excretion of sodium, potassium, and creatinine in normal rats after daily oral administration of distilled water, PPBE, and furosemide.

**Groups**		**Urinary electrolytes (mmol/L)**	**Urinary excretion (mmol/24 hr)**	**Creatinine (mg/L)**
	**Sodium**	**Potassium**	**Sodium**	**Potassium**	
**Control**	87.5±0.5	61.2±1.2	0.022±0.01	0.273±0.08	44.3±0.3
**PPBE D1-200 mg**	103.5±1.75^ ****++++####^	51±0.1^***+++####^	0.101±0.02^***^	0.511±0.61^***+###^	102.2±2.2^****++++####^
**PPBE D2-400 mg**	105±0.5^****++++####^	53.02±0.35^***+++####^	0.090±0.009^***^	0.505±0.033^**+^	97.7±5.3^****++++####^
**Furosemide**	124.8±0.2^****^	72.1±0.1^***^	0.082±0.01^***^	0.648±0.07^***^	53.4±1.6^*^

Data are expressed as mean± SEM; PPBE D1=Pinus. Pinaster bark extract dose 1 (200 mg/kg); PPBE D2= Pinus pinaster bark extract dose 2 (400 mg/kg); *Comparison between the control group and all groups. ***p<0.001; +Comparison between furosemide group and PPBE D1- 200 mg/kg. +++p<0.001; #Comparison between furosemide and PPE-D2 400mg group. #p<0.001

### Effect of oral administration of PPBE on plasma electrolytes and creatinine levels

Table 5 describes the impact of PPBE and furosemide on serum electrolyte levels. Rats treated with two doses (200 and 400 mg/kg) did not show any significant changes in plasma concentrations of potassium and sodium compared to the control group. However, the concentration of creatinine in the plasma was significantly reduced at the low dose of 200 mg/kg, with a value of around 3.11 ± 0.01 g/l compared to the control (p<0.001). The high dose of 400 mg/kg did not significantly affect plasma creatinine. On the other hand, furosemide significantly decreased plasma potassium levels (p<0.001) compared to the control group, the lower and higher dose of PPBE. 

### Effect of oral administration of PPBE on creatinine clearance

**Figure 3:**
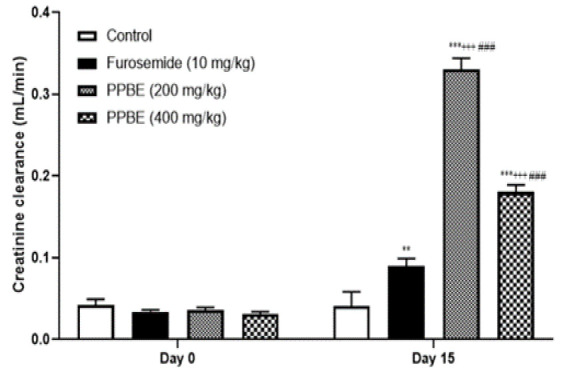
Creatinine clearance (mL/min) on plasma in normal rats after daily oral administration of PPBE, furosemide, and distilled water. Data are expressed as mean±SEM; PPBE D1= P. pinaster bark extract dose 1(200 mg/kg); PPBE D2= Pinus pinaster bark extract dose 2(400 mg/kg); *Comparison between the control group and all other groups ***p<0.001; +Comparison between furosemide group and PPBE D1- 200 mg/kg. +++p<0.001. #Comparison between furosemide group and PPE D2- 400 mg/kg. #p<0.001

On the final day of treatment, both PPBE and furosemide resulted in a significant elevation in creatinine clearance compared to the initial value and the control group. The increase was notably more pronounced in the PPBE-treated groups at different doses (200 and 400 mg/kg) than in the furosemide group, with the lower dose having the highest effect Figure 3).

### Effect of oral administration of PPBE on osmolarity and free water clearance

The present study revealed that both PPBE and furosemide led to an increase in osmolar clearance and urine osmolarity, accompanied by a decrease in free water clearance compared to the control group (p<0.001). However, there was no significant impact of PPBE or furosemide on plasma osmolarity (Table 6).

**Table 5:** Plasma concentration of creatinine, sodium, and potassium in normal rats after daily oral administration of PPBE, furosemide, and distilled water.

**Groups**		**Plasma electrolytes (mmol/l)**		**Creatinine (g/l)**
	Sodium		Potassium	
Control	136.03±2.03		5±0.5	5±0.5
PPBE D1-200 mg	138.33±0.67		4.8±0.02^++^	3.11±0.01^***+++##^
PPBE D2-400 mg	141±1.01		4.5±0.25^+^	5.15±0.85^#^
Furosemide	135.03±0.97		3.9±0.1^***^	5.1±0.1

Data are expressed as mean± SEM; PPBE D1=Pinus pinaster bark extract dose 1(200 mg/kg); PPBE D2= Pinus pinaster bark extract dose 2 (400 mg/kg); *Comparison between the control group and all other groups. ***p<0.001; +Comparison between furosemide group and PPBE D1- 200 mg/kg. +++p<0.001. #Comparison between furosemide group and PPE D2- 400 mg/kg. #p<0.001

**Table 6:** Impact of oral administration of PPBE, furosemide, and distilled water on urine osmolarity, plasma osmolarity, osmolar clearance, and clearance of free water on day 15.

Groups	Plasmatic osmolarity (mOsm/L)	Urinary osmolarity (mOsm/L)	Osmolar clearance (µl/min)	Free water clearance (µl/min)	Free water reabsorption (T_CH2O_) (µl/min)
**Control**	281.06±4.06	301.4±3.4	3.32±1.03	-0.20±0.01	0.20±0.01
**PPBE D1-200 mg**	285.66±1.34	313±3^***+++###^	7.62±0.86^***^	-0.68±0.17*^+++###^	0.68±0.17*^+++###^
**PPBE D2-400 mg**	291±2.02	320.4±0.6^***+++###^	7.26±0.41^***^	-0.65±0.07*^+++###^	0.65±0.07*^+++###^
**Furosemide**	279.06±1.94	397.8±0.6^***^	8.91±1.03^***^	-2.66±0.34^***^	2.66±0.34^***^

Data are expressed as mean±SEM; PPBE D1= Pinus pinaster bark extract dose 1 (200 mg/kg); PPBE D2= Pinus pinaster bark extract dose 2 (400 mg/kg) *Comparison between the control group and all other groups. ***p<0.001; +Comparison between furosemide group and PPBE D1- 200 mg/kg. +++p<0.001;#Comparison between furosemide group and PPE D2- 400 mg/kg. #p<0.001

## Discussion

The objective of this study was to examine the phytochemical constituents and assess the antioxidative and diuretic impacts of orally administered PPBE in Wistar rats. Pine, which has been a common plant in folk medicine remedies worldwide, has attracted considerable attention for its therapeutic potential. Different by-products from pine trees, particularly the bark, have been the focus of extensive exploration due to their abundant reservoir of phenolic compounds (Bhardwaj et al., 2021), which have proven important antioxidant activity. That plays a crucial role in protecting against oxidative stress (Toma et al., 2015). Polyphenols possess the potential to be utilized as a basis for developing new drugs.

The phytochemical screening of this research revealed that PPBE contained nine polyphenol compounds, including taxofoliol, gallic acid, catechin, epicatechin, ferulate glucoside, procyanidin B1, procyanidin B2, procyanidin B3, and proanthocyanidin. These results are aligned with those obtained by (Yesil-Celiktas et al., 2009), where four compounds are quantified using HPLC (epicatechin, catechin, taxifolin, and catechin gallate) in seven different pine species collected from both Germany (*P. ponderosa, P. nigra, P. parviXora, *and* P. sylvestris*) and Turkey (*P. sylvestris, P. nigra*, and* P. pinea*). In the same context, another investigation conducted by Vieito et al. (Vieito et al., 2019) identified gallic acid, taxifolin, ferulic acid, quercetin, and catechin in the phenolic profile of the hydroethanolic extract of *P. pinaster *bark collected from the northwest of Portugal.

The quantification assay of total polyphenol contents (TPC) and flavonoid contents (TFC) of PPBE showed a substantial quantity of phenolic compounds. The TPC concentration in our extract was around 34.11 ± 0.02 mg GAE/g DM. This result is consistent with those reported by Ferreira-Santos et al. (2019), which demonstrated the high level of TPC in the bark extracts of *P. pinaster *from the north region of Portugal. Furthermore, Chupin et al. (2013) examined the bark of *P. pinaster* from the southwest of France, and they discovered that the TPC levels ranged from 22 to 62 mg GAE/g DM. Our data are superior to those found in the studies by Chupin et al. (2015), with a mean value of TPC 28.30 mg GAE/g bark of *maritime pine* from the Landes forest. Flavonoids are a subclass of polyphenols that are particularly abundant in PPBE. Flavonoids, known for their antioxidant qualities, have been linked to several health advantages. The total flavonoid contents (TFC) in our extract were around 24.96±0.03 mg QE/g DM. The research conducted by Royer et al. (2013) showed that the hydroethanolic (95% ethanol) and aqueous extracts of Canadian pine species have TFC values between 6 and 39 mg quercetin equivalents/g extract. In addition, Sharma et al. (2015) derived hydroethanolic extracts (90% ethanol) with a high TFC concentration from the bark of three distinct pine species from India: *Pinus wallichiana*, *Pinus gerardiana*, and *Pinus roxburghii*. This rich and varied composition affects the antioxidant potential of PPBE and gives it a wide range of biological and pharmacologic properties.

Living systems continually produce free radicals, and an excess of these compounds can result in significant damage to tissues and biomolecules, potentially causing pathological conditions such as inflammation (Sharma et al., 2015) and impacting kidney function (Tucker et al., 2015). Utilizing antioxidants to scavenge free radicals may provide a defense against oxidative stress and help prevent cellular damage (Sharma et al., 2015). The antioxidant capacity of PPBE was measured by three different assays: DPPH, PR, and TAC. Our result provided interesting antiradical activity. The IC_50_ value of DPPH was 9.76±1.14 µg/ml, which is lower than those obtained by Ramos et al. (2022), with a mean value of IC_50 _= 22.53±0.46 μg/ml. The reducing power (RP) assay is also important, with an EC_50_ value of 0.037±0.004 mg/ml. This result is in line with those obtained by Apetrei et al. (2011) which show the strong reducing power of *Pinus cembra L*. bark value of EC_50 _= 0.026±0.3 mg/ml. The total antioxidant capacity (TAC) of our PPBE was very significant, with a value of 975.44±24.59 mg EAA/g DM, and it was higher than those obtained by Jiménez-Moreno et al. (Jiménez-Moreno et al., 2019).

The originality of the current research consists of being the first to evaluate the diuretic effect of PPBE in Morocco. Diuretic medications are helpful for several illnesses, such as liver cirrhosis, hypertension, and hypercalciuria. It is important to demonstrate their effectiveness in controlling electrolytes and water balance, given their therapeutic application in managing edema (Hailu and Engidawork, 2014). The diuretic potential of PPBE was studied in the present work in normal rats. Data obtained proved that PPBE affects positive diuretic function during daily dose administration over 15 days. 

PPBE is a source of essential minerals like potassium and magnesium, which are necessary for maintaining fluid balance and supporting kidney function. Our study indicated that PPBE is rich in these minerals and could potentially produce diuretic effects by influencing electrolyte balance. Both doses (200 and 400 mg/kg) of PPBE increased urinary output, urinary concentration of sodium, and decreased urinary potassium concentration compared with furosemide. This increase in sodium and water elimination could be beneficial in managing hypertension and edema diseases.

In general, diuresis is often defined by the following two phenomena: an increase in the excretion of water (urine output) and an increase in the elimination of electrolytes (Wile, 2012), similar to the action of furosemide. Furosemide enhances the rate of urine flow and promotes the excretion of sodium, potassium, and chloride. This is achieved through the inhibition of the Na+–K+–2Cl− symporter in the thick ascending loop of Henle and the suppression of carbonic anhydrase (Aissaoui et al., 2008). The reason that our extract does not cause a significant increase in potassium excretion such as furosemide is probably due to various mechanisms of action. Maybe PPBE is not specifically designed to target the sodium-potassium-chloride co-transporter. Their diuretic effects are mediated through other pathways.

Moreover, the overall impact of PPBE on potassium excretion may depend on various factors such as polyphenol compounds, dosage, duration of exposure, and individual variations in renal function. It is important to note that while furosemide is a potent diuretic, it can cause excessive potassium loss, leading to hypokalemia. This is why patients taking furosemide often require monitoring of their potassium levels and may need potassium supplementation (Oh and Han, 2015). The safe use of our extract is a general assumption because it is natural and does not pose a risk of causing severe potassium depletion.

The two doses did not affect the plasma sodium, or potassium, although furosemide decreased serum potassium significantly (p<0.001). As well the two doses caused an increase in creatinine clearance. These results show that our PPBE has a very strong effect on creatinine clearance, which reflects its ability to improve kidney function.

The administration of our PPBE increases water reabsorption and decreases free water clearance. It is important to consider the different mechanisms of renal physiology. The diuretic effect may be mediated by inhibiting sodium and chloride reabsorption in the renal tubules, increasing the osmotic pressure of the renal tubular fluid, and promoting diuresis (Wile, 2012). The dual action reflects the body's need to maintain homeostasis, while diuresis increases urine output to eliminate excess fluid or solutes. Compensation mechanisms can conserve water, especially when hydration is a priority (Cadwallader et al., 2010). Furthermore, the reduction in free water clearance indicates a selective effect on dissolved water reabsorption, possibly regulating the water permeability of renal tubular cells (Natochin et al., 2021). To support these ideas, it is crucial to study the specific bioactive compounds in the extracts and their effects on the kidneys. The study by Dizaye et al. (2013) showed that certain compounds in the extract can modulate renal function and influence renal blood flow, tubular reabsorption rates, or hormonal responses (Jahromi and Jahromi, 2020).

It is still unknown what active ingredient in *P. pinaster*'s hydroethanolic extract causes the diuretic and natriuretic effects. Qualitative phytochemical screening of PPBE demonstrated the presence of flavonoids, proanthocyanidins, and phenolic acids, which possess antioxidant and anti-inflammatory properties (Jurikova et al., 2018), can contribute to improved endothelial function in blood vessels (Behl et al., 2020) which leads to improved blood flow to the kidneys and may affect renal function, increasing urine production. These compounds have been studied for their potential effects on various health conditions, including urinary tract infections, bladder dysfunction, and benign prostatic hyperplasia (BPH) (Csikós et al., 2021). 

It is reasonable to suggest that these secondary metabolites may act individually or synergistically to produce the observed diuretic and natriuretic activities of *P. pinaster* bark. Previous research has shown the diuretic and natriuretic effects of these phytochemicals through a variety of mechanisms. For instance, flavonoids, natural antagonists of A1 adenosine receptors, can trigger diuresis and sodium (Na+) excretion either by directly blocking Na+ reabsorption in the proximal tubules or by indirectly causing dilation of the afferent arterioles. The antagonistic action on these receptors is commonly linked with diuretic effects (Jacobson et al., 2002). Adenosine A1 receptors play a crucial role in the proximal convoluted tubule (PCT), where they mediate the reabsorption of 60–70% of the filtered sodium and water (Welch, 2015). Therefore, antagonists of adenosine A1 receptors preserve the glomerular filtration rate by dilating the renal afferent arterioles, enhancing renal blood flow, promoting sodium excretion (natriuresis), and increasing urine production (diuresis) (Modlinger and Welch, 2003). Additionally, some other studies have investigated the effects of pine bark extracts, such as Pycnogenol, which is derived from *P. pinaster*. Pycnogenol has been shown to improve symptoms associated with BPH, including increased urinary flow rate and reduced frequency of urination (Ledda et al., 2018).

In summary, the findings from this study indicate that the bark of *P. pinaster *of Morocco is rich in polyphenols and flavonoids, contributing to its notable antioxidant activity. The administration of two doses of PPBE resulted in considerable enhancement in urine volume, creatinine clearance, urine creatinine, and urine sodium without producing hypokalemia, in comparison to furosemide. The high diuretic activity observed in this study appears to be closely linked to the significant antioxidant components, such as the phenolic compounds, found abundantly in our extract. However, additional research is required to isolate, purify, and determine the structure of the specific diuretic molecule responsible for this effect. It is also important to assess its safety, and long-term efficacy, and explore the potential mechanisms of action. 

## References

[1] Aissaoui A, El-Hilaly J, Israili ZH, Lyoussi B (2008). Acute diuretic effect of continuous intravenous infusion of an aqueous extract of Coriandrum sativum L in anesthetized rats. J Ethnopharmacol.

[2] Apetrei CL, Tuchilus C, Aprotosoaie AC, Oprea A, Malterud KE, Miron A (2011). Chemical, antioxidant and antimicrobial investigations of Pinus cembra L. Bark and Needles. Molecules.

[3] Ashmawy NA, Al Farraj DA, Salem MZM, Elshikh MS, Al-Kufaidy R, Alshammari MK, Salem AZM (2020). Potential impacts of Pinus halepensis Miller trees as a source of phytochemical compounds: Antibacterial activity of the cones essential oil and n-butanol extract. Agrofor Syst.

[4] Asmae EG, Nawal EM, Bakour M, Lyoussi B (2021). Moroccan monofloral bee pollen: Botanical origin, physicochemical characterization, and antioxidant activities. J Food Qual.

[5] Bakour M, Al-Waili NS, El Menyiy N, Imtara H, Figuira AC, Al-Waili T, Lyoussi B (2017). Antioxidant activity and protective effect of bee bread (honey and pollen) in aluminum-induced anemia, elevation of inflammatory makers and hepato-renal toxicity. J Food Sci Technol.

[6] Behl T, Bungau S, Kumar K, Zengin G, Khan F, Kumar A, Kaur R, Venkatachalam T, Tit DM, Vesa CM, Barsan G, Mosteanu D-E (2020). Pleotropic effects of polyphenols in cardiovascular system. Biomed Pharmacother.

[7] Bishaw B, Soolanayakanahally R, Karki U, Hagan E (2022). Agroforestry for sustainable production and resilient landscapes. Agrofor Syst.

[8] Braga ME, Santos RM, Seabra IJ, Facanali R, Marques MO, de Sousa HC (2008). Fractioned SFE of antioxidants from maritime pine bark. J Supercrit Fluids.

[9] Cadwallader AB, de la Torre X, Tieri A, Botrè F (2010). The abuse of diuretics as performance-enhancing drugs and masking agents in sport doping : Pharmacology, toxicology and analysis. Br J Pharmacol.

[10] Chupin L, Maunu SL, Reynaud S, Pizzi A, Charrier B, Charrier-EL Bouhtoury F (2015a). Microwave assisted extraction of maritime pine (Pinus pinaster) bark: Impact of particle size and characterization. Ind Crops Prod.

[11] Chupin L, Motillon C, Charrier-El Bouhtoury F, Pizzi A, Charrier B (2013b). Characterization of maritime pine (Pinus pinaster) bark tannins extracted under different conditions by spectroscopic methods, FTIR and HPLC. Ind Crops Prod.

[12] Csikós E, Horváth A, Ács K, Papp N, Balázs VL, Dolenc MS, Kenda M, Kočevar Glavač N, Nagy M, Protti M, Mercolini L, Horváth G, Farkas Á (2021). Treatment of benign prostatic hyperplasia by natural drugs. Molecules.

[13] Deghima A, Righi N, Rosales-Conrado N, León-González ME, Gómez-Mejía E, Madrid Y, Baali F, Bedjou F (2020). Bioactive polyphenols from Ranunculus macrophyllus Desf Roots: Quantification, identification and antioxidant activity. S Afr J Bot.

[14] Dizaye KF, Alberzingi BO, Sulaiman SR (2013). Renal and vascular studies of aqueous extract of Urtica dioica in rats and rabbits. Iraqi J Vet Sci.

[15] El Ghouizi A, El Menyiy N, Falcão SI, Vilas-Boas M, Lyoussi B (2020). Chemical composition, antioxidant activity, and diuretic effect of Moroccan fresh bee pollen in rats. Vet World.

[16] El Kamari F, Laaroussi H, Ousaaid D, El Atki Y, Taroq A, Aouam I, Lyoussi B, Abdellaoui A (2021). Diuretic effect of aqueous extracts of Vitex agnus castus leaves and seeds in Wistar Albinos rats. Int J Pharm Res.

[17] Farjon A (2001). World checklist and bibliography of conifers, 2nd edn. World checklists and bibliographies.

[18] Ferreira-Santos P, Genisheva Z, Botelho C, Santos J, Ramos C, Teixeira JA, Rocha CM (2020a). Unravelling the biological potential of pinus pinaster bark extracts. Antioxidants.

[19] Ferreira-Santos P, Genisheva Z, Pereira RN, Teixeira JA, Rocha CM (2019b). Moderate electric fields as a potential tool for sustainable recovery of phenolic compounds from Pinus pinaster bark. ACS Sustain. Chem Eng.

[20] Gupta S, Neyses L (2005). Diuretic usage in heart failure: A continuing conundrum. Invited Review.

[21] Hailu W, Engidawork E (2014). Evaluation of the diuretic activity of the aqueous and 80% methanol extracts of Ajuga remota Benth (Lamiaceae) leaves in mice. BMC Complement Altern Med.

[22] Hajhashemi V, Zolfaghari B, Amin P (2021). Anti-nociceptive and anti-inflammatory effects of hydroalcoholic extract and essential oil of Pinus eldarica in animal models. Avicenna J Phytomed.

[23] Jacobson KA, Moro S, Manthey JA, West PL, Ji X, B. S. Buslig (2002). Interactions of flavones and other phytochemicals with adenosine receptors. Flavonoids in Cell Function.

[24] Jahromi SM, Jahromi SN (2020). The effect of hydro-alcoholic extract of pumpkin seeds on estrogen levels and kidney markers in adult female rats. Iran Red Crescent Med J.

[25] Jerez M, Pinelo M, Sineiro J, Núñez MJ (2006). Influence of extraction conditions on phenolic yields from pine bark: Assessment of procyanidins polymerization degree by thiolysis. Food Chem.

[26] Jiménez-Moreno N, Volpe F, Moler JA, Esparza I, Ancín-Azpilicueta C (2019). Impact of extraction conditions on the phenolic composition and antioxidant capacity of grape stem extracts. Antioxidants.

[27] Jurikova T, Skrovankova S, Mlcek J, Balla S, Snopek L (2018). Bioactive compounds, antioxidant activity, and biological effects of european Cranberry (Vaccinium oxycoccos). Molecules.

[28] Klimánkova A (2007). Comparative study of Xavonoid contents and antioxidant activities of supercritical CO2 extracted pine barks grown in different regions of Turkey and Germany. Eur food Res technol.

[29] Lahlou S, Tahraoui A, Israili Z, Lyoussi B (2007). Diuretic activity of the aqueous extracts of Carum carvi and Tanacetum vulgare in normal rats. J Ethnopharmacol.

[30] Ledda A, Belcaro G, Feragalli B, Cornelli U, Dugall M, Corsi M, Cesarone MR (2018). Benign prostatic hypertrophy: Pycnogenol® supplementation improves prostate symptoms and residual bladder volume. Minerva Med.

[31] Mansouri S, Hosseini M, Beheshti F, Sobhanifar M-A, Rakhshandeh H, Anaeigoudari A (2021). Neuroprotective effects of Pinus eldarica in a mouse model of pentylenetetrazole-induced seizures. Avicenna J Phytomed.

[32] Mármol I, Quero J, Jiménez-Moreno N, Rodríguez-Yoldi MJ, Ancín-Azpilicueta C (2019a). A systematic review of the potential uses of pine bark in food industry and health care. Trends Food Sci Technol.

[33] Mármol I, Quero J, Jiménez-Moreno N, Rodríguez-Yoldi MJ, Ancín-Azpilicueta C (2019b). A systematic review of the potential uses of pine bark in food industry and health care. Trends Food Sci Technol.

[34] Modlinger PS, Welch WJ (2003). Adenosine A1 receptor antagonists and the kidney. Curr Opin Nephrol Hypertens.

[35] Natochin YuV, Shakhmatova EI, Bogolepova AE (2021). The mechanism of the solute-free water reabsorption increase in the rat kidney by oxytocin saluresis. Dokl Biochem Biophys.

[36] Ntchapda F, Bonabe C, Kemeta Azambou DR, Talla E, Dimo T (2016). Diuretic and antioxidant activities of the aqueous extract of leaves of Vepris heterophylla (Engl R Let (Rutaceae) in rats. BMC Complement Altern. Med.

[37] Oh SW, Han SY (2015). Loop diuretics in clinical practice. Electrolyte Blood Press.

[38] Packer L, Rimbach G, Virgili F (1999). Antioxidant activity and biologic properties of a procyanidin-rich extract from pine (Pinus maritima) bark, pycnogenol. Free Radic Biol Med.

[39] Plomion C, Chagne D, Pot D, Kumar S, Wilcox P, Burdon R, Prat D, Peterson D, Paiva J, Chaumeil P, Giovanni Giuseppe V, Sebastiani F, Nelson C, Echt C, Savolainen O, Kubisiak TL, Cervera MT, De María N, Faridi N (2007). The pines. Genome Mapping and Molecular Breeding in Plants.

[40] Ramos PAB, Pereira C, Gomes AP, Neto RT, Almeida A, Santos SAO, Silva AMS, Silvestre AJD (2022). Chemical characterization, antioxidant and antibacterial activities of Pinus pinaster Ait and Pinus pinea L Bark polar extracts: prospecting forestry by-products as renewable sources of bioactive compounds. Appl Sci.

[41] Rohdewald P (2002). A review of the french maritime pine bark extract (Pycnogenol), a herbal medication with a diverse clinical pharmacology. Int J Clin Pharmacol Ther.

[42] Roush GC, Kaur R, Ernst ME (2014). Diuretics : A review update. J Cardiovasc Pharmacol Ther.

[43] Royer M, Prado M, García-Pérez ME, Diouf PN, Stevanovic T (2013). Study of nutraceutical, nutricosmetics and cosmeceutical potentials of polyphenolic bark extracts from Canadian forest species. Pharmanutrition.

[44] Santos MC, Soares KD, Beltrame BM, Toson NSB, Do Carmo B Pimentel M, Bordignon SAL, Apel MA, Mendez ASL, Henriques AT (2021). Polyphenolic composition and in Vitro antihypertensive and anti‐inflammatory effects of cuphea lindmaniana Cuphea urbaniana. Chem Biodivers.

[45] Sharma A, Goyal R, Sharma L (2015). Potential biological efficacy of Pinus plant species against oxidative, inflammatory and microbial disorders. BMC Complement Altern Med.

[46] Singleton VL, Rossi JA (1965). Colorimetry of total phenolics with phosphomolybdic-phosphotungstic acid reagents. Am J Enol Vitic.

[47] Toma C-C, Olah N-K, Vlase L, Mogoșan C, Mocan A (2015). Comparative studies on polyphenolic composition, antioxidant and diuretic effects of Nigella sativa L (black cumin) and Nigella damascena L (lady-in-a-mist) seeds. Molecules.

[48] Tucker PS, Scanlan AT, Dalbo VJ (2015). Chronic kidney disease influences multiple systems: describing the relationship between oxidative stress, inflammation, kidney damage, and concomitant disease. Oxid Med Cell Longev.

[49] Vieito CDS, Pires P, Fernandes É, Vaz Velho M (2019). Chemical characterization of pine bark (Pinus pinaster Aiton subsp Atlantica) antioxidant properties and phenolic profile of its extracts. Millennium.

[50] Vivar-Quintana AM, González-Martín MI, Revilla I, Betances-Salcedo EV (2018). Determination and quantification of phenolic acids in raw propolis by reversed phase high performance liquid chromatography: Feasibility study for the use of near infrared spectroscopy. J Apic Res.

[51] Welch WJ (2015). Adenosine, type 1 receptors : Role in proximal tubule Na + reabsorption. Acta Physiologica.

[52] Wile D (2012). Diuretics : A review. Ann Clin Biochem.

[53] Willcox JK, Ash SL, Catignani GL (2004). Antioxidants and prevention of chronic disease. Crit Rev Food Sci Nutr.

[54] Xie Q, Liu Z, Li Z (2015). Chemical composition and antioxidant activity of essential oil of six pinus taxa native to China. Molecules.

[55] Yesil Celiktas O, Isleten M, Vardar-Sukan F, Oyku Cetin E (2010). In vitro release kinetics of pine bark extract enriched orange juice and the shelf stability. Br Food J.

[56] Yesil-Celiktas O, Otto F, Parlar H (2009). A comparative study of flavonoid contents and antioxidant activities of supercritical CO2 extracted pine barks grown in different regions of Turkey and Germany. Eur. Food Res Technol.

[57] Zuluaga DCM, Serrato BJC, Quicazán de CMC (2014). Valorization alternatives of colombian bee-pollen for its use as food resource-a structured review. Vitae.

